# The Dosage Effect of Laser Acupuncture at PC6 (Neiguan) on Heart Rate Variability: A Pilot Study

**DOI:** 10.3390/life12121951

**Published:** 2022-11-22

**Authors:** Yi-Chuan Chang, Chun-Ming Chen, Ing-Shiow Lay, Yu-Chen Lee, Cheng-Hao Tu

**Affiliations:** 1 Graduate Institute of Acupuncture Science, College of Chinese Medicine, China Medical University, Taichung 404328, Taiwan; 2 Department of Chinese Medicine, China Medical University Beigang Hospital, Yunlin 651012, Taiwan; 3 Department of Medical Imaging, China Medical University Hospital, Taichung 404333, Taiwan; 4 School of Post Baccalaureate Chinese Medicine, China Medical University, Taichung 404328, Taiwan; 5 School of Chinese Medicine, China Medical University, Taichung 404328, Taiwan; 6 Department of Chinese Medicine, China Medical University Hospital, Taichung 404328, Taiwan

**Keywords:** laser acupuncture, energy density, heart rate variability, Neiguan, autonomic nerve system

## Abstract

Laser acupuncture (LA) has been more applicated in the clinical practice with good responses, but the dosage and parameter settings are still inconsistent with the arguments. This study is focused on the effect of LA on heart rate variability (HRV) with different energy density (ED). Based on the Arndt–Schulz law, we hypothesized that the effective range should fall within 0.01 to 10 J/cm^2^ of ED, and settings above 10 J/cm^2^ would perform opposite or inhibitory results. We recruited healthy adults in both sexes as subjects and choose bilateral PC6 (Neiguan) as the intervention points to observe the HRV indexes changes by an external wrist autonomic nerve system (ANS) watch on the left forearm. The data from the ANS watch, including heart rate, blood pressure, and ANS activity indexes, such as low frequency (LF), high frequency (HF), LF%, HF%, LF/HF ratio, and so on, were analyzed by the one-way ANOVA method to test the possible effect. In this study, every subject received all three different EDs of LA in a randomized order. After analyzing the data of 20 subjects, the index of HF% was upward and LF/HF ratio was downward when the ED was 7.96 J/cm^2^. Otherwise, the strongest ED 23.87 J/cm^2^ performed the opposite reaction. Appropriately, LA intervention could affect the ANS activities, with the tendency to increase the ratio of parasympathetic and decrease the ratio of sympathetic nerve system activities with statistically significant results, and different ED interventions are consistent with Arndt–Schulz law with opposite performance below and above 10 J/cm^2^.

## 1. Introduction

Laser acupuncture (LA), one of the applications of low-level laser therapy (LLLT), has been used more and more recently in clinic because of its non-invasive, non-thermal, painless, and no bleeding or blood stasis characteristics, rather than traditional acupuncture to stimulate specific regions or acupoints [1,2]. The advantages of LA also include flexible adjustable exposed zones, accurate dosing of the exposure, nonspecific patterns of receptor structures, and the ability to combine the technique with any type of treatment [1]. Acupuncture practitioners have combined the characteristics of LA with traditional Chinese medicine (TCM) theory and acupuncture intervention principles in treating diseases for several years with good responses. There have been more publications on the application and efficacy of LA, including for the problems in which traditional acupuncture is difficult or only limited in intervention, which improves the functional outcomes after total knee replacement [3], carpal tunnel syndrome (CTS) symptoms [4], long-term unrecovered facial palsy [5], pain reducing and increasing motion in temporomandibular disorders patients [6], spasticity relieve of cerebral paralysis children [7], and speech and social interaction performance in autism children [8]. In other words, the physiological mechanisms of LA and LLLT are likely to be the same or similar to some extent. These forms of phototherapy, or photobiomodulation, use photons to produce a photochemical effect by mitochondria physiology reaction [9,10], and have reported several known physiological effects, including stimulating healing, reductions in inflammation, pain relief, promotion of circulation, and regeneration of tissues [11]. However, although LA has a wide range of applications and flexible operation, different models or parameter settings may have different effects, and the measures used vary between studies.

One published paper suggested to control the energy density (ED) between 4 to 10 J/cm^2^, because the ineffective studies appeared more often due to over-dosing than to under-dosing [12]. In the early years, some authors considered that LA could not produce the expected effect, which may also be related to this reason [13]. Furthermore, a review article indicated that different radiant exposure of ED dosage may have different biological effects; ED around 10 J/cm^2^ may have different or opposite biological reactivity [14]. For example, some studies have pointed out that LA tends to increase sympathetic nerves and inhibit parasympathetic nerves in patients with insomnia [15], but others have found the opposite performance in LA interventions for night shift worker groups by different LA intervention and parameter settings [16]. To sum up the above, the interventional effect of LA may be affected by different dosages, or ED, and have different biological effects, which means they may have a biphasic dosage effect.

Heart rate variability (HRV) is a noninvasive technique for monitoring changes in the cardiac cycle for assessing the autonomic activity [17]. The indexes from HRV measurement contain time domain and frequency domain data. The time domain data include heart rate (HR), systolic blood pressure (SBP), diastolic blood pressure (DBP), standard deviation of normal–normal intervals in msec (SDNN), root mean square of the sum of squares of normal–normal intervals differences (RMSSD), and proportion of the difference between normal–normal exceeding 50 ms (PNN50). Time domain data, including RR-interval and variance, could be analysis and Fourier transformation to execute frequency domain analysis [18]. Frequency domain data contain total power (TP), very low frequency power (VLF; 0.003~0.04 Hz), low frequency power (LF; 0.04~0.15 Hz), high frequency power (HF; 0.15~0.4 Hz), low frequency percentage (LF%), high frequency percentage (HF%), and LF/HF ratio. The index of HF is usually regarded as the indicators of parasympathetic system (PNS) activities; however, the index of LF is regarded as the indicators of sympathetic activities with parasympathetic influence, and the index of sympathetic system (SNS) power is expressed as the LF/HF ratio [18].

HRV indexes are not only the indicators of neuro-cardiac function that reflects heart–brain interactions and autonomic nervous system (ANS) dynamics, but also can be regarded as one of the reference indicators of individual physiological or pathological changes. Systematic review articles reported that HRV biofeedback is a potential intervention to improve fine and gross motor function in athletes [19,20]. Similar principles of HRV have also been widely used in daily life, such as sports bracelets or watches. There are also more publications on the relationship between diseases and HRV. The manifestations of parasympathetic withdrawal and sympathetic over-activity have been discussed in much of the literatures in major depressive disorder groups [21]. Furthermore, altered HRV indexes were shown to be related to cardiovascular risk, including sudden cardiac risk, or in some neurological diseases, such as diabetes, stroke, multiple sclerosis, muscular dystrophies, Parkinson’s disease, and epilepsy [22]. In high cardiovascular risks groups, including post-myocardial infarction patients and heart failure patients, low HRV has been shown to be independently predictive of increased mortality of sudden death [23]. That is to say, the measurements and changes of HRV can respond to the individual’s physiological and pathological characteristics and, furthermore, may have a predictive role at some level or as a basis for comparison before and after.

In TCM and acupuncture applications, the Neiguan (PC6) acupoint is widely used for treating symptoms of heart and chest diseases, such as palpitations, chest pain, nausea, or vomiting [24]. Much experimental and clinical evidence has reported that acupuncture at PC6 improved heart function, including vessel dilation, and could reduce the area of damage in myocardial infarction [25,26]. Following acupuncture intervention at PC6 in patients with angina pectoris, cardio-angiographic measurements of coronary diameters have revealed dilation in the obtuse marginal branch in patients [25]. Moreover, electroacupuncture pretreatment at bilateral PC6 and Ximen (PC4) could significantly reduce the likelihood of post-percutaneous coronary intervention myocardial injury in patients with coronary artery disease [26,27]. Certainly, PC6 stimulation has also been proven to lower HR, SBP, suppress the enhanced SNS activities with LF/HF ratio decreases, and increase PNS activities with a higher HF power [27,28]. Much of the evidence above shows that proper stimulation of PC6 should have empirically proven effects to affect the functional performance of the heart and ANS activities. However, there is still no clear consensus or any relevant articles discussing how to achieve the expected response by LA, and there is not enough empirical evidence for the different responses that different dosages may produce.

Our aim for this study is to use LA for intervention to examine the biological reactivity of different dosages and mainly focus on ED at PC6. According to Arndt–Schulz law, we hypothesize that the ED below and above 10 J/cm^2^ may induced different or opposite changes. Further, to test our hypothesis, after providing different ED in bilateral PC6, we combined with the measurement of HRV to examine the effects on the ANS. Through analyses of the changes of different indexes in HRV, we expect that the changes of SNS and PNS activities and the relative activity percentage to examine the changes in ANS after PC6 intervention could be observed.

## 2. Materials and Methods

### 2.1. Ethics Approval

The implementation of this clinical trial program abides by the Declaration of Helsinki, stipulated by the World Medical Association, so as to ensure the life and safety of clinical trial subjects’ health, personal privacy, and dignity. In addition, this research project had passed the clinical trial/human research review of the Research Ethics Committee of China Medical University and Affiliated Hospital on 17 September 2021, case number: CMUH110-REC2-151.

### 2.2. Participants

We recruited healthy subjects who aged among 18 to 55 y/o with BMI among 18.5 to 24 kg/m^2^ in both sexes. Considering the risk factor of coronary artery disease (CAD), postmenopausal female subjects were excluded, since it is a risk factor of CAD [29]. Considering the safety of experiments and the stability of data collection, the other exclusion criteria were as follows: (1) subjects diagnosed with chronic diseases, such as CAD, hypertension (HTN), diabetes mellitus (DM), hyperlipidemia, and cancer history; (2) subjects with CTS symptoms or syndromes recently; (3) subjects with functional or organic diseases associated with CNS; (4) subjects cannot follow instructions or obey orders for the study; (5) pregnant women or subjects with presence of metal implants or pacemaker; (6) medicine associated with nerve activity inhibition, such as psychiatric medicines or analgesic drugs, in the past 3 months; (7) opening wound or scar around PC 6 area, which may interfere the intervention of LA.

### 2.3. Study Design

In this study, subjects received LA stimulation with 3 different EDs, 0, 7.96, and 23.87 J/cm^2^ included, according to the drawing order, and received about 5 min pre- and post-stimulation HRV measurements, respectively. Data such as LF%, HF%, LF/HF ratio, and so on were used as stimulus related to HRV change indexes. Owning to previous study, it was recommended that supine position would be a more suitable posture to detect HRV [30]; in our study, all subjects were lying in supine position, instead of sitting or other postures, for measurement to reduce the impact of unnecessary movement to data collection. Before the pre-HRV measurement, subjects should lie still for 15 min to measure in the awake resting-state before the execution of the study and avoid the surrounding environment interference. The purpose of this procedure is to establish physiological baseline as a benchmark for analysis and comparison to reduce the differences in HRV and ANS between individuals as much as possible. Furthermore, the former and the latter interventions should be separated by at least 5 min to avoid the influence of carry-over effect. All interventions were carried out under the same external environmental conditions and space.

### 2.4. Laser Acupuncture Intervention

The LA equipment used in this project is RJ laser (Handylaser Trion; Reimers and Janssen GmbH, Winden, Germany). The basic parameters were set in Bahr 2 oscillatory/resonant frequency, with pulsed-mode irradiation in 4 mm probe with 3 different stimulation EDs formed, including a control group with no actual energy output and two experimental groups with 7.96 and 23.87 J/cm^2^ for stimulation. The whole details of LA parameter settings are shown in Table 1.

**Figure 1:**
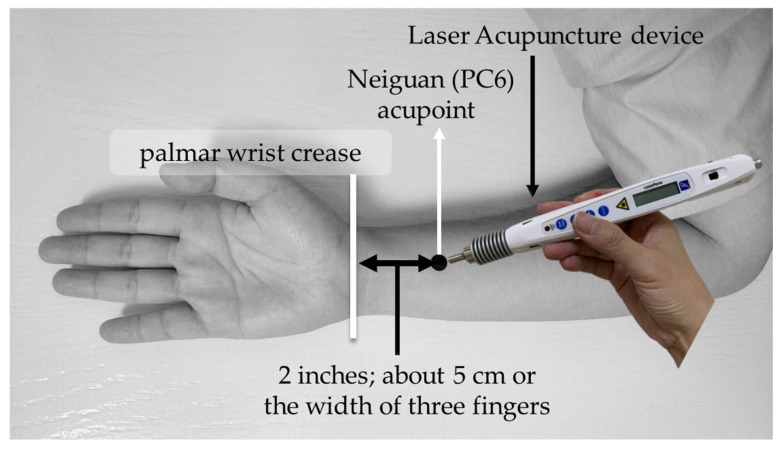
Schematic diagram of intervention and acupoint selection of PC6.

LA was performed on bilateral PC6 acupoints. The acupoint selection method of PC6 was based on the WHO standardized acupuncture point localization guidelines: selected at two inches above the transverse crease of the wrist, about 5 cm or the width of three fingers, between the palmar longus muscle and the flexor carpi radialis tendon [31]. The schematic diagram of intervention and acupoint selection of PC6 is shown in Figure 1. Moreover, in this study, LA operations were performed by qualified LA expert practitioner who had been well-trained with 8 years in TCM and more than two years of clinical experience in LA.

### 2.5. HRV Data Collection

For HRV changes of data measurements before and after intervention of different EDs, this study used the external wrist ANSWatch^®^ as the measurement tool (TS-0411 type, Taiwan Scientific Ltd., New Taipei CityTaiwan, which had been approved by ISO 13485, and EU CE Mark). The principle of ANSWatch^®^ is to use left radial artery as the medium to collect the pulsatile signals by oscillation with high sensitivity sensors and to perform further analysis and application through Fourier transform. In this study, the indexes collected from ANSWatch^®^ contained SBP, DBP, HR, HRV, RMSSD, PNN50, TP, VLF, LF, HF, LF%, HF%, and LF/HF ratio.

### 2.6. Statistical Analysis

All indexes from LA intervention with different ED of pre- and post-stimulation HRV measurements were subtracted and converted by percentage, and these changes between different EDs were analyzed by one-way ANOVA test, and the Tukey test was used for post-hoc analysis. A *p*-value of <0.05 was considered to be statistically significant.

## 3. Results

### 3.1. Subjects

We recruited 23 subjects, with 1 subject under body mass index (BMI) and 2 subjects above BMI acceptance criteria ruled out, with total 20 healthy subjects included in the final group. Among them, there were 13 female subjects and 7 male subjects. The subjects aged 30 to 39 accounted for most, 11 subjects in total, accounting for 55%; the BMI distribution of subjects was relatively average, 20.0 to 21.4 kg/cm^2^ accounted for most, a total of 7 people, accounting for 35%. The remaining basic information of the subjects, including gender, age, and BMI distribution, is shown in Table 2 for details.

### 3.2. Time Domain Indexes

In the time domain indexes, including SBP, DBP, and HR, the data before and after intervention in each group showed a slight downward trend in the values, but none of them with a statistically significant difference. Moreover, the remaining indexes, including HRV, RMSSD, and PNN50, which are associated with the variations of pulse intervals, still showed no statistically significant difference, although part of the indexes, such as HRV, were converted by percentage conversion, with more trends of statistical differences. The details of time domain indexes are shown in Table 3, and the results of percentage conversion are presented in italic.

### 3.3. Frequency Domain Indexes

In the frequency domain indexes, indexes related to ANS activities, including LF, HF, VLF powers, and TP, both the raw data after intervention and converted by percentage conversion in different ED, showed various changes but with no statistically significant difference, although part of them, such as LF, VLF powers, and TP, showed more trends of differences. In the index of TP’, we excluded the part of the VLF power because of the lesser amount to define the physiological meaning [18]; however, both the raw data and transformed by percentage conversion in each different ED still showed no statistically significant differences in TP’. Furthermore, the results presented in the percentage conversion showed the trends of changes to neural activities to be observed with the increase of ED, but there were no statistically significant differences. The results, in detail, of the indexes mentioned above are shown in Table 4.

**Figure 2:**
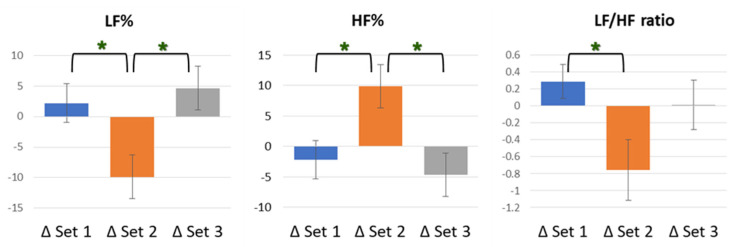
Schematic diagram of LF%, HF%, and LF/HF ratio analysis results. In LF%, Set 2 showed a downward trend with, respectively, statistical differences from Set 1 (*p* = 0.041) and Set 3 (*p* = 0.011). In HF%, Set 2 showed an upward trend with same statistical results. In LF/HF ratio, Set 2 showed a downward trend, with statistical difference from Set 1 (*p* = 0.040). There was no significant difference in the comparison of other indexes between groups. * *p* < 0.05.

LF% and HF% represent the respective percentage performance of LF and HF in the whole after the Fourier transformation of the subject’s own pulse interval various. In the index of LF%, ED 7.96 J/cm^2^ showed a downward trend, compared to other groups, with a statistically significant difference (*F* [2, 59]  =  5.137, *p* = 0.009), as well as, respectively, statistical differences from ED 0 J/cm^2^ (*p* = 0.041) and ED 23.87 J/cm^2^ (*p* = 0.011). However, data of ED 0 J/cm^2^ and ED 23.87 J/cm^2^ did not exist inter-groups differences. HF%, as the data corresponding to LF%, showed an upward trend and had the same statistical results, with significance. In the index of LF/HF ratio, ED 7.96 J/cm^2^ remained, showing a downward trend, compared to other groups, with an overall statistically significant difference (*F* [2, 59]  =  3.578, *p* = 0.034), as well as a statistical difference from ED 0 J/cm^2^ (*p* = 0.040). However, data from neither ED 0 J/cm^2^ and ED 23.87 J/cm^2^ nor ED 7.96 J/cm^2^ and ED 23.87 J/cm^2^ existed for inter-group differences in the index of the LF/HF ratio. The rest of the remaining details of frequency domain indexes are shown in Table 4, and the results of the percentage conversions are presented in italics. In addition, the HRV indexes with statistically significant differences, LF%, HF%, and LF/HF ratio, between groups are shown in Figure 2.

## 4. Discussion

In our study, we found out that an ED of 7.96 J/cm^2^ can cause an increase in the PNS and a decrease in the SNS performance, while the stimulation of the bilateral PC6 showed the opposite response when the ED reached 23.87 J/cm^2^. As far as we know, this study is the first research and explore the possible biphasic photobiomodulation effect of LA, based on different EDs. The results of Set 2 with ED 7.96 J/cm^2^, lower than 10 J/cm^2^, are consistent with Arndt–Schulz law and meet our hypothesis, with the tendency to increase the ratio of PNS with HF% enhanced and suppress SNS with LF/HF declined with statistically significant difference to Set 1. On the contrary, Set 3 was the strongest ED of all the settings with ED 23.87 J/cm^2^, over than 10 J/cm^2^, with the opposite tendency of changes to decrease the ratio of PNS with HF% declined and increase the SNS with LF/HF enhanced. It is also consistent with the hypothesis of study, despite the fact that the analysis results of 23.87 J/cm^2^ did not reach statistical differences with the others. Our preliminary results indicated that the stimuli PC6 with LA do affect HRV, and different ED of LA may produce effects in different directions.

Certainly, ED is not the only parameter that affects interventional responses. According to the previous research, it is suggested that the use of power density would be better less than 100 mW/cm^2^ and ED between 4 to 10 J/cm^2^ [12]. The energy output of our study design was identical in Set 2 and Set 3, with 398 mW/cm^2^, which was still beyond the recommendation, with post-intervention responses. The ED of Set 2 was 7.96 J/cm^2^, which met the parameters recommended, and the ED of Set 3 was 23.87 J/cm^2^, which was beyond the parameters recommended and partially consistent with our hypothesis of the study and the Arndt–Schulz law, and over-dosing of the stimulation may produce opposite or less than expected results. Actually, the absorption of the laser energy is different for certain regions, depending on the tissue composition and mitochondrial content of the stimuli regions [12]. In fact, studies using higher ED had also been reported with satisfactory results for targeting deeper tissues, such as bone, with 20-140 J/cm^2^ ED for new bone formation [32,33]. Moreover, for both in vivo and in vitro studies, the intervention results of different dosages were still inconclusive [12], not to mention the different effects of LA between different species. This is also one of the main reasons for the lack of a fixed mode of clinical use of LA, with difficulty to approve research.

HRV is generated by heart–brain interactions, dynamic non-linear ANS processes, and reflex neuro-cardiac functions [34]. The non-invasive, cost-effective, and straightforward characteristics of HRV index measurements are one of the reasons of being used for clinical research and very suitable as a medium for observation [35]. Practically, the measurement of HRV is also interfered with by many factors. Studies have shown that the difference in the measurement region and the environment temperature can affect the results [17]. Respiration, measurement posture, physical activity, and emotional stress have also been reported to affect the results [34]. In our study, all subjects were measured, and intervention in the same indoor environment with the same humidity and temperature in supine position reduced the interference caused by extrinsic factors. Although the subjects in this study were all healthy subjects, with excluded cardiovascular disease, HTN, DM, hyperlipidemia, and cancer history, there may still other factors potentially causing individual differences between the subjects that could not be perfectly controlled.

According to the results of this study, by HRV monitoring and indexes analysis in healthy subjects, stimulating bilateral PC6 with an ED of 7.96 J/cm^2^ can, indeed, increase HF%, which is an indicator of PNS, and decrease the LF/HF ration, which represents an indicator of SNS. These results are in line with the treatment principles of TCM acupuncture and indicate that the proper stimulation of PC6 in health people may activate the PNS and modulate the balance of ANS, which probably has the effect of preventive health care for some symptoms or diseases related to SNS over-activation. Although we did not incorporate the observations of pathological changes of HRV for specific disease or preventive intervention in this study. To some extent, HRV detection can not only reflect the effect of LA intervention, but also means that HRV may be used as a reference or intervention tracking index for related clinical symptoms. As mentioned above, HRV could reflect not only the physiological but also pathological state and disease or even prognosis of people. The relationship between disease and HRV has also been explained in many ways. Reduced HRV overall activities and LF levels were observed in patients with bipolar disorder, compared with healthy control individuals [36], and more depressive episodes or longer durations of manic or hypomanic episodes were also associated with lower HRV [37]. In children with autism spectrum disorder, subjects exhibited significantly lower HRV and HF levels [38]. For moderate to severe traumatic brain injury patients, TP, HF power, and LF/HF ratio were statistically significant for predicting mortality [39]. Low levels of HF have also been shown to be associated with suicidal ideation and behavior in students and depressed patients, rather than non-suicidal controls [40]. Woman with interstitial cystitis/bladder pain syndrome had lower vagal activity in index of HF, when compared with healthy controls, as confirmed with a shift towards sympathetic dominance in index of LF/HF ratio [41]. The HRV variables had the association with Parkinsonian motor symptom duration, with a more affected cardiac parasympathetic of HF than sympathetic regulation [42]. Although the physiologic basis for VLF power is far less clear than HF and LF power [43], VLF is receiving increasing attention, as it may be associated with heart disease. VLF power is regarded as an independent risk predictor in patients with congestive heart failure [44] and was the strongest predictor of the prognostic after acute myocardial infarction and mortality than HF or LF power [33,45]. Masked HTN is also reported to relate to the LF/HF ratio increasing significantly [46]. A decreased LF/HF ratio also demonstrated a worse outcome in vessel occlusion patients who received thrombectomy under general anesthesia [47]. In recent years, HRV has been applied as an indicator to predict COVID-19 prognosis, with higher HRV predicting greater chances of survival, especially in patients aged 70 years and older and low HRV predicts ICU indication and admission in the first week after hospitalization [48].

Although our study still has many shortcomings, it makes three important contributions. First, as far as we know, this article is the first article that combines LA intervention and HRV detection to speculate the various effects with different ED. The utilization of LA may be affected by different models, parameter settings, and regulation, which may produce different effects. Based on Arndt–Schulz Law, different dosages of ED may exist a biphasic dose-response phenomenon [3,14], and our preliminary results confirmed this viewpoint, with 10 J/cm^2^ as the dividing point, which is partially consistent with the arguments announced by previous studies, to control ED under 4 to 10 J/cm^2^ for the target tissue [12,14]. Second, our research also combines the principle of TCM in the study design to choose bilateral PC6 as the intervention region. In the theory of TCM and acupuncture, PC6 is a classic acupoint to use in clinical settings, which is generally considered to have the effect of activating the PNS for treating symptoms or diseases, such as improved gastric problems [49], reduce fatigue [50], lower blood pressure [28,51], and decline the HR of atrial fibrillation subjects [51]. Overall, stimuli PC6 is provided with the role of activity modulation in the cardiovascular system, with an effect to attenuate sympatho-excitatory cardiovascular reflex responses [52]. Third, we integrated the concepts of the first and the second viewpoints above, and our preliminary results revealed how to provide appropriate energy stimulation, ED in this study, so that the stimulation of PC6 may produce corresponding effects, similar to the general clinical purpose, and propose a direction to follow.

However, some limitations should be noted. From the initial stage of data collection and the results, we realize that the neural activity of subjects varies greatly among each index, although some of the reasons may be related to the influence of the measurement method. Additionally, there is known literature pointing out that HRV measurement and variations may be affected by different ages [53], genders [54], and different measurement times [55]. In view of this, we use specific age and BMI as the screening conditions for healthy subjects and measure the HRV before each LA intervention to establish a baseline to further the follow-up and analysis to minimize the possible impact of the aforementioned reasons as much as possible, after all, it is difficult to require all subjects to intervene at the same time and in the same space context. Furthermore, there are many variables that can be manipulated in the clinical application of LA. In addition to the wavelength, probe size, power, and irradiation time mentioned above, different pulse emission oscillatory/resonant frequencies are also important parameters for clinical use. In our study, Bahr 2 frequency is selected as the emission frequency, but the effects caused by different frequencies are rarely discussed in the known journals. Therefore, although the preliminary results of this study indicated that LA could have some influence on ANS, it is uncertain whether the use of other frequencies will have similar or different results. Numerous studies have shown that there are significant differences in HRV or neural activity between healthy and unhealthy individuals [56,57,58]. Therefore, although the dosage and frequency of interventions used in this study can respond, whether they can affect other groups with significant responses or varying degrees of changes are unknown. Finally, the literature also reported that different breathing patterns will also have varying degrees of impact on HRV [59,60]. However, in the actual execution of the experiment, asking subjects to pay attention to breathing or to adjust breathing patterns may interfere with the experiment and prevent subjects from remaining in a relatively relaxed state. There is no better way than to establish a baseline first to reduce the differences of the breathing patterns between individuals. What is more, in addition to the variability that may be caused by breathing, even though we all require subjects to stay in an awakened resting-state, whether or not the subjects can actually follow the instructions to avoid excess brain activity is also a limitation we cannot control, and the potential impact on the measurement results is also unknown.

## 5. Conclusions

LA is one of the emerging treatment media that has been gradually valued and widely used in recent years, but the empirical medical data presented in the current literature still have many imperfections. In this study, we figured out that LA stimulation does affect ANS, and different EDs may produce various physiological reactions. According to the changes in the indexes, the preformation of PNS activity upward and SNS activity downward was observed when the ED was below 10 J/cm^2^ after bilateral PC6 stimulation. However, although we found that appropriate energy stimulation with LA can have a corresponding effect on ANS, more and wider research is still needed in the future for further exploration. We also hope that the traditional acupuncture, with a thousand-year-old history, can develop a new milestone by means of LA.

## Figures and Tables

**Table 1:** Parameters of the laser device with different intervention Sets of the groups.

**Laser device**		Gallium-aluminum-arsenide (GaAIAs) infrared laser RJ laser				
**Wavelength**		810 nm				
**Intervention mode**		Contact				
**Frequency**		Pulsed wave at Bahr 2: 1199 Hz for central/middle tissue layer				
**Intervention region**		Bilateral PC6				
**Set**	**Probe** **Size**	**Output** **Power**	**Exposure** **Time**	**Single** **Joule**	**Total** **Joule**	**Energy** **Density**
1	4 mm	0 mW	20 s	0 J	0 J	-------------
2	4 mm	100 mW	20 s	1 J	2 J	7.96 J/cm^2^
3	4 mm	100 mW	60 s	3 J	6 J	23.87 J/cm^2^

**Table 2:** Demographic of the study population.

Sex	
Female	13
Male	7
**Age, Years**	
<20	0
20–29	7
30–39	11
40–49	2
>50	0
Mean ± SD	32.25 ± 5.52
**BMI (kg/cm^2^)**	
18.5–19.9	4
20.0–21.4	7
21.5–23.0	4
23.0–24.0	5
Mean ± SD	21.48 ± 1.60

Abbreviation: BMI, Body Mass Index; SD, standard deviation.

**Table 3:** Heart Rate variability (HRV) statistical analysis results of time domain indexes.

Index	Group, Mean ± SD			*p*-Value			
	Δ Set 1 0 J/cm^2^	Δ Set 2 7.96 J/cm^2^	Δ Set 3 23.87 J/cm^2^	Over All	Set 1 vs. 2	Set 1 vs. 3	Set 2 vs. 3
**SBP** (mmHg)	−3.6 ± 5.73	−3 ± 4.34	−2.1 ± 4.69	0.631	0.923	0.607	0.834
**DBP** (mmHg)	−1.5 ± 2.72	−0.45 ± 2.63	−0.55 ± 1.61	0.311	0.348	0.420	0.990
**HR** (bpm)	−0.6 ± 3.91	−0.45 ± 4.11	−1.55 ± 4.30	0.658	0.993	0.746	0.676
**HRV ^§^** (ms)	−6 ± 30.95 *2% ± 0.4*	−4.3 ± 41.72 *13% ± 0.94*	3.1 ± 56.33 *35% ± 0.95*	0.788 *0.416*	0.992 *0.895*	0.793 *0.394*	0.857 *0.666*
**RMSSD**	−3.9 ± 30.98	2 ± 34.42	5.8 ± 39.10	0.679	0.855	0.657	0.937
**PNN50** (%)	−4 ± 23.27	0.1 ± 25.83	5.05 ± 28.45	0.564	0.886	0.535	0.819

**^§^** Percentage conversion of the index, in italics; Abbreviation: SD, standard deviation; SBP systolic blood pressure; DBP, diastolic blood pressure; HR, heart rate; HRV, heart rate variability, which means variations of R(Normal)–R(Normal) intervals in msec; RMSSD, root mean square of the sum of squares of R(Normal)–R(Normal) intervals; PNN50, proportion of the difference between R(Normal)–R(Normal) intervals exceeding 50 ms.

**Table 4:** Heart Rate variability (HRV) statistical analysis results of frequency domain indexes.

Index	Group, Mean ± SD			*p*-Value			
	Δ Set 1 0 J/cm^2^	Δ Set 2 7.96 J/cm^2^	Δ Set 3 23.87 J/cm^2^	Over All	Set 1 vs. 2	Set 1 vs. 3	Set 2 vs. 3
**LF power ^§^** (ms^2^)	−232.3 ± 2122.34 *67% ± 2.13*	−653.1 ± 2532.79 *127% ± 4.96*	855.95 ± 3606.65 *510% ± 12.87*	0.227 *0.178*	0.885 *0.979*	0.447 *0.200*	0.218 *0.297*
**HF power ^§^** (ms^2^)	−230.45 ± 977.17 *33% ± 1.31*	68.20 ± 1134.10 *121% ± 3.86*	422.4 ± 1320.80 *294% ± 7.67*	0.209 *0.254*	0.963 *0.846*	0.182 *0.235*	0.598 *0.522*
**VLF power ^§^** (ms^2^)	−766.8 ± 3099.65 *24% ± 1.63*	−8.05 ± 3946.00 *131% ± 5.05*	−1240.95 ± 486.85 *167% ± 4.53*	0.788 *0.509*	0.907 *0.679*	0.962 *0.504*	0.773 *0.957*
**TP ^§^** (ms^2^)	−1229.6 ± 5617.16 *19% ± 0.94*	−592.95 ± 6764.02 *113% ± 4.79*	37.4 ± 10750.78 *168% ± 3.59*	0.883 *0.403*	0.966 *0.678*	0.872 *0.377*	0.967 *0.871*
**TP’ ^§^** (ms^2^)	−462.75 ± 2967.77 *49% ± 1.62*	−337.05 ± 3431.80 *121% ± 4.43*	1278.35 ± 4729.40 *390% ± 10.42*	0.276 *0.236*	0.994 *0.937*	0.320 *0.240*	0.374 *0.408*
**LF%**	2.2 ± 14.01	−9.85 ±15.88	4.65 ± 15.97	0.009 **	0.041 *	0.869	0.011 *
**HF%**	−2.2 ± 14.01	9.85 ±15.88	−4.65 ± 15.97	0.009 **	0.041 *	0.869	0.011 *
**LF/HF** **ratio**	0.278 ± 0.91	−0.756 ± 1.63	0.107 ± 1.29	0.034 *	0.040 *	0.910	0.103

**^§^** Percentage conversion of the index, in italics; Abbreviation: SD, standard deviation; LH, low frequency; HF, high frequency; VLF, very low frequency; TP, total power, which means the summation of LH, HF, and VLF power; TP’, total power’, which means the summation of LH and HF power. * *p* < 0.05, ** *p* < 0.01.

## Data Availability

All data analyzed in this study are available from the corresponding author on reasonable request.
